# Prosocial Behavior and Contributors to Older Adults’ Well-Being in Daily Life

**DOI:** 10.1093/geroni/igaa057.2080

**Published:** 2020-12-16

**Authors:** Jeanne Nakamura, Dwight Tse

## Abstract

This symposium reports findings from a national experience-sampling study of 165 older adults (mean age=71, range=60-88 years) who are heavily involved in prosocial activity, contributing to their communities as leaders or high-commitment volunteers in social-purpose organizations. Gerontological research has linked prosocial activity to a set of positive outcomes for the older adults who engage in it (e.g., better health and reduced mortality), including global measures of well-being such as life satisfaction. However, little is known about the contributors to these individuals’ momentary well-being. Four presentations address this gap in knowledge. In the first presentation, Dwight Tse revisits the concept of successful aging as a within-person variable with day-to-day variations. Experience sampling data revealed great variations in successful aging indicators, and successful aging was associated with better well-being as hypothesized. In the second presentation, Ajit Mann extends beyond general control beliefs and explores the diversity in distribution of control beliefs across various daily activities (e.g., active leisure, prosocial activity, socializing, etc.), in addition to investigating the relationship between later life control diversity and subjective well-being. In the third presentation, Kelsey Finley explores Aristotle’s concept of the golden mean (an ideal state between deficiency and excess) for the number of hours spent in prosocial work on momentary well-being outcomes. In the fourth presentation, Jeanne Nakamura examines the relationship between global meaning in life and meaningful engagement in the moment, illuminating variation in this relationship through a comparison of the prosocial leaders and high-commitment volunteers.

